# The Association between Overweight and Illegal Drug Consumption in Adolescents: Is There an Underlying Influence of the Sociocultural Environment?

**DOI:** 10.1371/journal.pone.0027358

**Published:** 2011-11-16

**Authors:** Francesca Denoth, Valeria Siciliano, Patricia Iozzo, Loredana Fortunato, Sabrina Molinaro

**Affiliations:** Institute of Clinical Physiology, Italian National Research Council – CNR, Pisa, Italy; The University of Queensland, Australia

## Abstract

**Background:**

The aims of the study were to: a) Examine the distribution of gender-stratified body mass index (BMI), eating attitudes and use of addictive substances, under the hypothesis of a confluent prevalence of weight abnormalities, eating disorders and substance abuse. b) Demonstrate the extent to which family, peer-related and psychosocial factors are common elements in categories of compulsive behaviour.

**Methodology/Principal Findings:**

In the present cross-sectional study, data were collected through self reported questionnaires administered to a large sample of 33,185 15–19 years old adolescents (ESPAD®Italia), divided into weight categories based on the BMI percentile distribution. Multinomial analyses were adopted to address the influence of social, family, leisure time factors, Eating Attitude Test (EAT26) on the association between weight categories and drug use.

Recent drugs use was more frequent in overweight and underweight adolescents (p<0.05), especially in females. An EAT26 score ≥20 was more common in overweight adolescents. Multinomial analysis abolished the relationship between overweight and the use of most drugs, implicating self-esteem, parents' educational level, and friendships as mediators of the association. Within the overweight category, adolescents reporting recent drug use, showed greater frequency of having drug-abusing friends (∼80%), and severe problems with parents and school (∼30%) compared to overweight adolescents without recent drug use.

**Conclusion:**

The frequent association of overweight and substance use and the presence of common underlying social factors, highlights the need for an interdisciplinary approach involving individual-focused treatment models as well as public health, social and environmental changes to reduce food- and substances-related problems.

## Introduction

Obesity is a major public health problem, and its prevalence is growing at an alarming rate, especially in children and adolescents. Italy is one of the European countries with the highest number of overweight youth [1]. The hypothesis that obesity implicates food addiction, thus occurring in association or as an alternative to other substance abuse, is attracting attention [2]. Overeating in obese individuals shares similarities with loss of control and compulsive drug-taking behaviour observed in drug-addicted subjects, and people suffering from an eating disorder may also exhibit other addictive behaviours, including alcoholism and drug use. Though studies using functional or metabolic brain imaging may suggest innate alterations in neural circuits controlling reward seeking in subjects with drug addiction, eating disorders and obesity [3], it is important to keep in mind that eating attitudes and self-image perception are greatly dependent on cultural background and social pressure. Both eating disorders and drug addiction may indicate low self-esteem, at least partialy modulated by the subject's interaction with peers and parents. Eating disorders such as bulimia and anorexia are most commonly found in the upper social classes of industrialized countries, in which being slim reinforces the feeling of social acceptance; immigration from non-industrialized to industrialized countries is associated with an increase in the incidence of abnormal eating attitudes, underlining the importance of the acquired component in the modulation of eating behaviours [4].

Adolescents are an important target of investigation and prevention in this area. First, weight and eating abnormalities in early life are likely carried over into adulthood, since approximately one-third (26–41%) of preschool, half (42–63%) of school-age obese children, and up to 70% of obese adolescents become obese adults [5]. Second, the early appearance of obesity entails the prolonged exposure to related risk factors. Third, drug addiction starts at this age, and teenagers are also extremely susceptible to psychosocial influences.

Defining the strength of the association between obesity, eating disorders and use of addictive substances, and understanding whether these disorders have similar underlying psychosocial environment is important in identifying modifiable risk elements (e.g self-esteem, relations with parents, friends, etc.) that can and need to be targeted in preventive and treatment programmes.

The aims of the latest nationwide survey (2007) in a large sample of Italian adolescents aged 15–19 years were to a) examine the distribution of gender-stratified body mass index, eating attitudes and use of potentially addictive licit and illicit substances, under the hypothesis of a confluence of weight abnormalities, eating disorders and substance abuse, b) demonstrate the extent to which family, peer-related and educational psychosocial factors are common elements in extreme categories of compulsive behaviour in adolescents.

## Methods

### Recruitment

Sampling and data collection procedures are summarized here; full details are available in the 2007 European School Survey Project on Alcohol and Other Drugs (ESPAD) Report [6]. Data collection was performed by standardized methodology using anonymous self-administered questionnaires completed in the classroom, participation was completely voluntary. None of the students refused to participate in the study. The response rate of schools participating was 92.4%. The target population comprised Italian high-school students aged 15–19 years. After the compilation of the standard ESPAD2007 questionnaire, students were asked to fill in an additional module, including self-reported body weight and height, and the Eating Attitude Test 26 [7]. Of 41,470 participants, 33,815 (n = 16,904 males, n = 16,911 females) consistently responded to the additional module.

### Dependent variable: body mass index (BMI)

We defined as overweight (OW) those adolescents who were above the 90th percentile, underweight (UW) those under the 10^th^ percentile, and normal-weight (NW) those ≥10^th^ percentile and ≤90^th^ percentile of the BMI distribution in each age category [8] (males: UW 1,642; NW 13,452; OW 1,810 - females: UW 1,709; NW 13,515; OW 1,687).

### Independent variables

Personal variables included: age, gender and EAT26 score.

Satisfaction with self- and interpersonal relationship variables included: engaging often with friends for fun, receiving care from parents and/or friends, being satisfied with the relationship with parents and/or friends, being satisfied with the family's financial situation, being satisfied with one's own health and self, having experienced serious problems with parents, friends and/or teachers, having friends who abused drugs or alcohol.

Family variables included: parents' schooling level, family's financial situation, parental monitoring, family structure (living with both parents or not).

Substance use or health-compromising behaviour variables included: having used substances during the last year and last month (tranquillizers or sedatives without a prescription, cannabis, cocaine, heroin, stimulants, hallucinogens and dieting drugs), binge drinking ≥3 times in the last month, having smoked ≥11 cigarettes per day during the last month, gambling (e.g. slot machine), having spent more than €50 per week without parental control, having been in trouble with the police, or having engaged in regrettable sexual intercourse.

School variables included: having missed one or more classes for ≥3 days during the last month without a suitable reason, having obtained high marks in the last term.

Leisure time variables are divided into sedentary and non-sedentary activities. Sedentary activities included: playing videogames almost every day, reading books for pleasure, other hobbies (e.g. playing an instrument), watching TV more than 4 hours/day. Non-sedentary activities included: going out in the evening (e.g. to a disco), actively participating in sports.

### Statistical analysis

Students' social background, attitudes and drug use were summarized by gender for the three groups (underweight, normal-weight, overweight), by using percentages. Statistical analyses were performed with Stata 9.2. The Chi-square test was first used to compare each variable in the three groups (stratified by gender). The statistically significant variables (p <.05) from the above univariate analyses were included in the multinomial regression analysis, Normal-weight was used as the reference category. Given the large number of variables taken into account, similar to the classification of Dominé [8] and Janssen [9], we divided the independent variables in two sets: the first focusing on the psychosocial traits and the second concerning leisure and school activities. Two multinomial regression models were performed in order to analyze the role played by these groups of variables in the association between BMI categories and substance use. Model 1 includes personal, substance use and health-compromising behaviours variables and satisfaction with interpersonal relationships, self and family variables; while Model 2 includes personal, substances use, health-compromising behaviours and variables concerning school and leisure time. Results are reported as adjusted Odds Ratio (OR) and 95% confidence interval. Males and females were analyzed separately. Finally, we isolated the subgroup of subjects carrying both overweight and drug use traits, and re-examined socio-cultural and family structure in these high-risk individuals as compared with similarly overweight adolescents with a negative history of recent intake of drugs.

## Results

**Figure 1:**
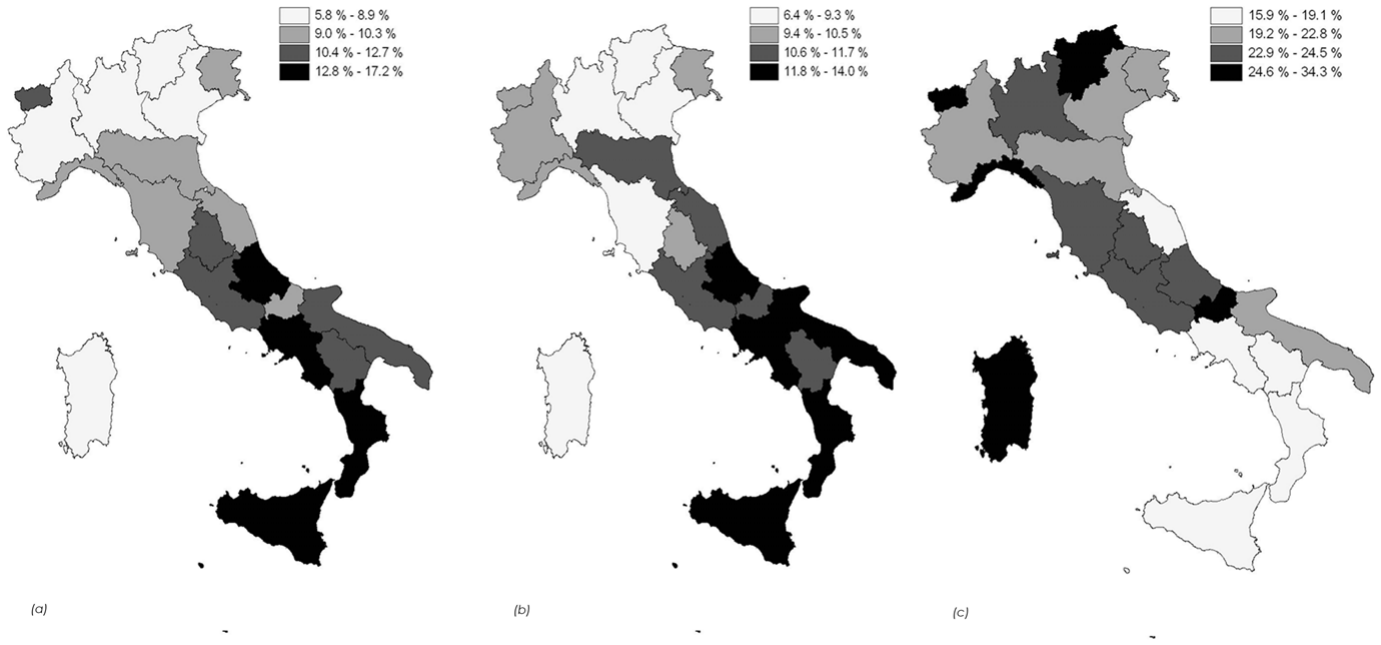
Geographical distribution of overweight (a), EAT26 >20 (b), substance use (c), expressed as percentage.

The prevalence of overweight and of EAT26≥20 in adolescents was higher in Central (10.3% and 10.5%, respectively) and Southern Italy and Islands (13.1% and 12.3%) than in Northern Italian Regions (8.6% and 8.7%). The occurrence of the use of an illegal substance, at least once in the last 12 months, was greater in Northern and Central Italy (∼22.5%) than in Southern Regions and Islands (20.7%); (Fig. 1a-c). The prevalence of Italian adolescents reporting a BMI ≥30 was 1.8% and higher in males than in females (respectively 2.7% and 1.0%).

### Univariate analysis

Table 1 shows the distribution of the independent variables in the weight- and gender-stratified categories. Normal-weight adolescents were satisfied with their health compared to peers belonging to the other weight categories.

**Table 1:** prevalence of characteristics among weight categories and gender.

CHARATERISTICS	MALE %				FEMALE %			
USE OF SUBSTANCES OR HEALTH COMPROMISING BEHAVIOURS	Underweight	Normalweight	Overweight	pvalue	Underweight	Normalweight	Overweight	pvalue
LM tranquillizers/sedative (no doctor's prescription)	0.6	0.5	0.7	n.s.	1.2	0.7	1.8	***
LM cocaine	0.6	0.4	0.7	n.s.	0.3	0.2	0.5	*
LM heroine	0.4	0.3	0.9	**	0.2	0.2	0.5	*
LM stimulants	0.4	0.6	0.8	n.s.	0.7	0.2	0.5	**
LM hallucinogens	0.8	0.4	0.9	**	0.5	0.2	0.5	**
LM drugs for dieting	0.1	0.2	0.2	n.s.	0.3	0.3	1.3	***
LM binge drinking (>3 times)	16.3	18.8	21.9	***	12.1	9.5	11.6	***
LY heroine	2.0	1.2	1.9	**	1.9	0.9	1.8	***
LY cannabis	23.2	26.4	22.9	***	20.4	18.7	17.7	n.s.
LY cocaine	4.2	4.9	4.1	n.s.	4.9	2.7	3.2	***
LY stimulants	3.0	3.5	3.1	n.s.	3.9	1.8	2.2	***
LY hallucinogens	4.1	3.0	2.7	*	2.9	1.5	2.3	***
LY drugs for dieting	0.9	0.8	2.4	***	1.6	3.3	7.0	***
cigarettes (>11/day)	23.9	25.7	29.3	***	29.9	27.5	31.7	***
play for money often	12.3	13.9	16.4	**	2.9	2.0	4.4	***
spend >50/week Euros without parents control	10.5	11.1	13.4	*	9.4	6.1	7.3	***
troubles with police	9.4	10.6	10.9	n.s.	5.7	4.2	5.0	**
regrettable sexual experiences	11.0	12.9	13.6	n.s.	11.3	9.6	12.0	**

SATISFACTION WITH SELF-AND INTERPERSONAL RELATIONSHIP	Under weight	Normal weight	Over weight	pvalue	Under weight	Normal weight	Over weight	pvalue
satisfied with one's own health	86.2	89.0	82.8	***	81.5	82.5	74.5	***
satisfied with him/herself (good self esteem)	77.0	80.9	74.0	***	70.2	67.9	57.9	***
satisfied with relationship with mother	84.0	84.3	87.0	**	76.1	76.5	75.8	n.s.
satisfied with relationship with friends	85.2	88.1	85.8	***	86.2	86.2	82.4	***
satisfied with the family financial situation	67.5	68.2	66.6	n.s.	61.1	61.2	56.1	***
go out with friends often	80.6	83.3	78.6	***	81.5	80.1	74.8	***
get warmth from friends	70.9	74.1	69.9	***	82.8	83.2	78.4	***
perceive the attention of parents	78.2	78.8	80.9	n.s.	83.8	86.4	85.6	*
troubles with parents	17.5	17.7	19.0	n.s.	26.9	26.6	30.3	**
troubles with friends	19.6	18.9	20.4	n.s.	25.3	24.2	28.2	***
troubles with teachers	20.2	22.3	22.4	n.s.	24.0	22.9	26.8	**
have friends who abuse alcohol and drugs	63.9	67.1	63.5	***	64.4	65.2	61.6	**

LEISURE TIME	Under weight	Normal weight	Over weight	pvalue	Under weight	Normal weight	Over weight	pvalue
play videogames almost every day	54.6	51.3	55.6	***	25.4	24.3	31.6	***
watch TV (>4 hours/day)	16.9	16.6	20.6	***	17.4	17.2	21.3	***
read book for pleasure almost every day	10.8	8.2	8.5	**	14.4	16.0	19.0	***
practice hobbies often	24.8	20.9	23.8	***	24.1	24.3	29.1	***
practice sports almost every day	32.8	44.6	32.1	***	19.5	23.2	18.8	***
go out in the evening often	66.6	74.2	68.1	***	72.4	70.1	60.0	***

SCHOOL	Under weight	Normal weight	Over weight	pvalue	Under weight	Normal weight	Over weight	pvalue
last 30 days skipping school in (>3 days)	15.7	14.6	15.5	n.s.	12.5	11.0	16.5	***
have a medium-high school performance	91.6	92.9	90.9	**	95.2	96.0	93.5	***

FAMILY	Under weight	Normal weight	Over weight	pvalue	Under weight	Normal weight	Over weight	pvalue
have a non traditional family	19.0	16.7	20.1	***	20.3	16.9	19.8	***
high financial family situation	89.8	92.0	88.8	***	89.4	90.1	86.0	***
High level of schooling- father	51.6	49.5	42.0	***	49.8	46.6	34.0	***
High level of schooling- mother	55.8	52.0	45.3	***	51.3	49.3	39.0	***
EAT-26 SCORE ≥20	3.9	3.6	11.3	***	10.8	14.5	24.3	***

For the use of substances, only significant variables are reported:

LM: use of the specific substance in the last 30 days (>10 times); LY: use of the specific substance in the last 12 month;

*: p<0.05;

**: p<0.01;

***: p<0.001; ns: p≥0.05.

In both sexes, living in a non-traditional family was associated with a greater likelihood of overweight and underweight. Higher education of either parent was associated with underweight. Smoking cigarettes, using dieting drugs, gambling, having a worse school performance, and a higher EAT26 score were more common in the overweight category.

In relation to gender, self-perception was most satisfactory in normal-weight males and underweight females. The likelihood of going out often with friends followed the same distribution. Rewards from the maternal relationship was associated with overweight in boys, but not girls. Overweight girls were characterized by lower self-esteem and a worse relationship with peers, parents and teachers; consequently, they missed days of school without specific reason. Both overweight and underweight girls reported regrettable sexual intercourse. The choice of drugs in the last 12 months was also gender-related, cannabis use was more strongly associated with normal-weight males, whereas cocaine use was more related to underweight females. Overweight boys and underweight girls experienced more binge drinking and spent ≥€50 per week without parental control. Sedentary activities were more frequent in underweight and overweight boys, and overweight girls.

### Multinomial analysis

#### Model 1

Family structure and finances, self-perception and esteem, relationships with friends (Table 2).

**Table 2:** Family structure and conditions, self perception and esteem, relationship with friends: under and overweight adolescents vs normal weight adolescents.

	MALE				FEMALE			
ASSOCIATIED FACTORS	OR (95% CI)Under weight	pvalue	OR (95% CI)Over weight	pvalue	OR (95% CI)Under weight	pvalue	OR (95% CI)Over weight	pvalue
LY cannabis	0.818 (0.682;0.982)	*	0.706 (0.590;0.846)	***	n.s.		n.s.	
LY cocaine	0.680 (0.462;1.000)	n.s.	0.614 (0.413;0.914)	**	1.881 (1.372;2.580)	***	0.896 (0.598;1.341)	n.s.
LY stimulants	n.s.		n.s.		n.s.		n.s.	
LY hallucinogens	2.448 (1.686;3.553)	***	0.953 (0.604;1.503)	n.s.	n.s.		n.s.	
LY drugs for dieting	0.763 (0.344;1.694)	n.s.	2.402 (1.466;3.933)	***	0.413 (0.251;0.680)	***	1.655 (1.262;2.171)	***
LM binge drinking (>3 times)	0.823 (0.685;0.988)	*	1.199 (1.016;1.415)	*	1.241 (1.018;1.512)	*	1.288 (1.049;1.581)	*
cigarettes (>11/day)	1.014 (0.851;1.209)	n.s.	1.471 (1.248;1.733)	***	n.s.		n.s.	
have friends who abuse alcohol and drugs	n.s.		n.s.		0.956 (0.839;1.09)	ns	0.82 (0.717;0.936)	**
have a non traditional family	n.s.		n.s.		1.189 (1.014;1.394)	*	1.054 (0.889;1.249)	n.s.
medium-high financial family situation	0.738 (0.596;0.913)	**	0.828 (0.669;1.025)	n.s.	n.s.		n.s.	
satisfied with one's own health	0.911 (0.737;1.126)	n.s.	0.663 (0.545;0.805)	***	0.823 (0.695;0.974)	*	0.769 (0.654;0.904)	**
satisfied with him/herself (good self esteem)	0.876 (0.739;1.038)	n.s.	0.784 (0.665;0.925)	**	1.132 (0.980;1.307)	ns	0.716 (0.622;0.825)	***
satisfied with relationship with mother	1.062 (0.888;1.269)	n.s.	1.447 (1.198;1.749)	***	0.999 (0.862;1.158)	ns	1.226 (1.052;1.429)	**
high level of schooling-father	1.029 (0.893;1.186)	n.s.	0.801 (0.694;0.924)	**	1.121 (0.980;1.283)	ns	0.662 (0.572;0.766)	***
high level of schooling-mother	1.183 (1.026;1.364)	*	0.956 (0.829;1.101)	n.s.	1.023 (0.894;1.170)	ns	0.828 (0.718;0.954)	**
go out with friends often	0.908 (0.757;1.089)	n.s.	0.714 (0.602;0.848)	***	1.163 (0.963;1.404)	ns	0.721 (0.612;0.849)	***
get warmth from friends	0.921 (0.799;1.062)	n.s.	0.875 (0.761;1.006)	n.s.	0.971 (0.823;1.147)	ns	0.843 (0.721;0.987)	*
EAT-26 score ≥20	0.999 (0.700;1.426)	n.s.	2.885 (2.289;3.635)	***	0.687 (0.564;0.838)	***	1.523 (1.304;1.779)	***

LM: use of the specific substance in the last 30 days; LY: use of the specific substance in the last 12 month;

*: p <0.05;

**: p <0.01;

***: p <0.001; ns: p ≥0.05.

In male adolescents, independent positive relationships were maintained between overweight and cigarette smoking, use of anorexic products during the last year, frequent binge drinking in the last month, EAT26 score ≥20 and satisfaction with maternal relationships, while negative independent associations were observed between overweight and the cannabis or cocaine use during last year, satisfaction with their health or themselves, going out often with friends and having a father with high educational level. Underweight males were more frequent users of hallucinogens during the last year, had lower economic status and a mother with high educational level, were less prone to the use of cannabis and to binge drinking.

In overweight girls, independent positive relationships were maintained with use of drugs for dieting, binge drinking, and EAT26 score ≥20, while negative associations included having friends that used drugs, being satisfied with health and themselves, going out often with friends, having parents with high educational level, receiving emotional support from friends, and being satisfied with maternal relationships. Underweight females were more prone to use cocaine and to binge drinking, had a non-traditional family, reported a less frequent use of dieting products and EAT26 score ≥20, and greater satisfaction with their own health.

#### Model 2

School and leisure time (Table 3). In boys, independent associations were confirmed between overweight and the habit of spending money without parental control; they played videogames often and watched TV 4 hours or more/day. They were less likely to have participated in sports or have gone out in the evening. In girls, independent associations were confirmed between overweight and the habit of playing videogames, missing school frequently because they skipped; overweight girls were less likely to have good school grades, and to go out in the evening. Underweight females had spent money without parental control and underweight males and females were less likely to participate in sports, similar to respective overweight counterparts.

**Table 3:** School and leisure time: under and overweight adolescents vs normal weight adolescents.

	MALE				FEMALE			
ASSOCIATIED FACTORS	OR (95% CI)Under weight	pvalue	OR (95% CI)Over weight	pvalue	OR (95% CI)Under weight	pvalue	OR (95% CI)Over weight	pvalue
LY cannabis	0.883 (0.739;1.055)	n.s.	0.692 (0.579;0.827)	***	n.s.		n.s.	
LY cocaine	0.709 (0.485;1.035)	*	0.651 (0.442;0.959)	*	2.062 (1.525;2.787)	***	0.960 (0.653;1.413)	n.s.
LY stimulants	n.s.		n.s.		n.s.		n.s.	
LY hallucinogens	n.s.		n.s.		n.s.		n.s.	
LY drugs for dieting	0.777 (0.350;1.721)	n.s.	2.465 (1.524;3.987)	***	0.384 (0.225;0.654)	***	1.690 (1.28;2.232)	***
LM binge drinking (>3 times)	0.843 (0.702;1.0120)	n.s.	1.224 (1.037;1.444)	*	n.s.		n.s.	
cigarettes (>11/day)	1.017 (0.856;1.210)	n.s.	1.370 (1.163;1.613)	***	n.s.		n.s.	
spend>50/week euros without control	0.987 (0.799;1.219)	n.s.	1.245 (1.030;1.504)	*	1.638 (1.303;2.059)	***	1.072 (0.822;1.397)	n.s.
play videogames almost every day	1.173 (1.036;1.328)	*	1.227 (1.083;1.390)	**	1.094 (0.948;1.263)	n.s.	1.454 (1.268;1.669)	***
practice sports almost every day	0.603 (0.529;0.687)	***	0.562 (0.493;0.641)	***	0.776 (0.661;0.911)	*	0.758 (0.644;0.892)	**
go out in the evening often	0.742 (0.645;0.854)	***	0.751 (0.652;0.865)	***	1.107 (0.956;1.281)	n.s.	0.577 (0.503;0.661)	***
practice hobbies often	1.113 (0.963;1.285)	n.s.	1.157 (1.002;1.335)	*	0.976 (0.840;1.135)	n.s.	1.189 (1.030;1.373)	n.s.
watch TV (>4 hours/day)	1.000 (0.848;1.177)	n.s.	1.194 (1.023;1.393)	*	n.s.		n.s.	
last 30 days skipping school in (>3 days)	n.s.		n.s.		1.050 (0.859;1.282)	n.s.	1.67 (1.395;1.999)	***
take care of the house	n.s.		n.s.		0.960 (0.808;1.14)	n.s.	1.244 (1.032;1.5)	n.s.
have a medium-high school performance	n.s.		n.s.		0.832 (0.617;1.12)	n.s.	0.674 (0.510;0.893)	n.s.
EAT-26 score ≥20	0.986 (0.700;1.388)	n.s.	2.907 (2.322;3.639)	***	0.693 (0.565;0.85)	***	1.684 (1.437;1.973)	***

LM: use of the specific substance in the last 30 days; LY: use of the specific substance in the last 12 month;

*: p <0.05;

**: p <0.01;

***: p <0.001; ns: p ≥0.05.

**Figure 2:**
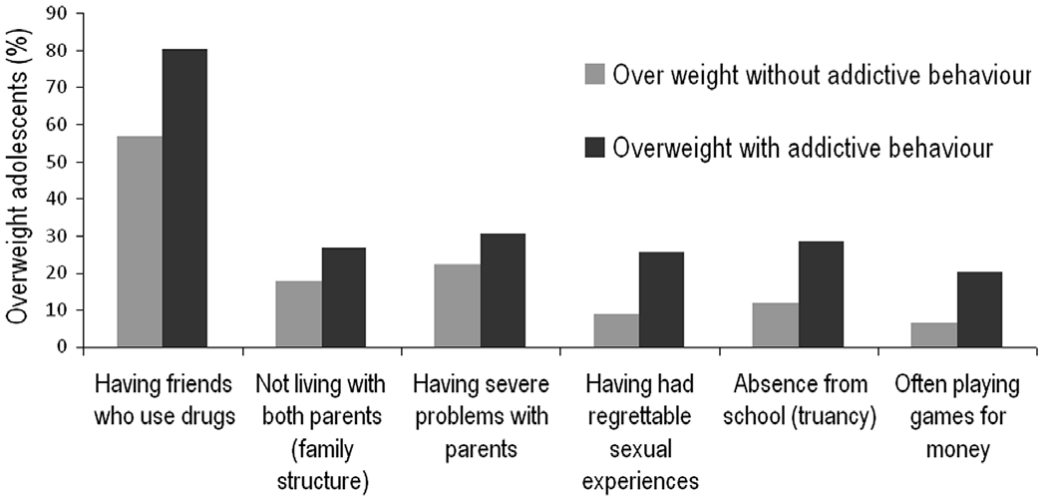
Prevalence of social, familiar and personal maladaptive traits among over weight adolescents with co-existing substance use, compared to adolescents with over weight condition alone.

Based on the results of the two multinomial models, an additional descriptive analysis was performed focusing only on adolescents with co-existing overweight and drug use (Fig. 2). This subgroup, compared to those with overweight alone, showed pronounced differences, including greater peer drug consumption, unconventional family structure, negative sexual experiences and serious problems with parents and school.

## Discussion

This study emphasizes the alarming severity of the current spread of the use of illicit substances and non-prescribed psychoactive drugs in adolescents. We examined a population of 33,815 high-school students, in whom the use of questionnaires, as opposed to face-to-face interviews, was instrumental to the broadness of the sample, and to a superior reliability, since it has been documented that participants feel more anonymous before a questionnaire [10]. BMI from self-reported height and weight has been shown not to affect the associations in epidemiologic studies [11]. The EAT26 has been widely used in assessing the risk of eating disorders, and has been validated in the Italian population [12].

Overall, our sample documented a recent use of cannabis in 22%, and of other addictive substances in 12% of these young people. In a significant proportion of 2% of subjects, the frequency of use was ≥10 times in the month preceding our survey. More than half of adolescents declared that they had friends abusing (not just using) alcohol or drugs. The current numbers are consistent with those reported in other countries. In the United States there has been an abrupt increase in illicit drug use in adolescents, starting from 1990, peaking in 1997 and doubling from adolescents to young adults [13], with frequencies of 21.9% and 22.3% in 2007 and 2008 respectively, which are superimposable to the ones observed in our 2007 survey [14]. Also, the use of non-prescribed drugs has recently increased among adolescents [15].

This prevalence allocates illegal drug use in adolescents within the scale of an epidemic of at least the same order of magnitude as the one described in the literature concerning obesity in this age range.

### How strong is the link between weight management, eating disorders and use of addictive substances?

Our data indicate that compared with their normal-weight peers, both underweight and overweight adolescents consumed more drugs, except for cannabis, and more tranquillizers and sedatives, without a medical prescription. Considering all drug categories, excluding cannabis, occasional use (at least once in the last 12 months) was higher in males than in females. In fact 7.6% underweight and 7.4% overweight boys, reported an occasional use vs 6.8% underweight and 5.3% overweight girls. Similarly frequent use (≥10 times in the last month) was more typical in males, involving 1.3% underweight and 1.7% overweight boys, than females (∼1.2 both in underweight and overweight).

Abnormal weight control is therefore associated with a 20–40% greater use and abuse of drugs. Both the more occasional and the regular use of substances coincided with a less satisfactory financial family situation in over- and underweight categories, in spite of an attitude of spending more money. In overweight but not in underweight categories there was also elevated use of dieting products, and a predisposition to eating disorders. The EAT26 score was in general greater in females than males, as unanimously reported in the literature, and it exceeded the cut-off of 20 in 2–3 times as many overweight than normal-weight adolescents. A similar ratio was seen in the use of dieting drugs. Previous studies have emphasized the relationship between obesity and EAT26 outcomes [16] and the need for an integrated medical approach to eating disorders and obesity [17]. Eating disorders are characterized by a series of personality traits, including compulsivity [18], and relationships between eating disorders and drug abuse have been previously shown [19], [ 20]. More specifically, several authors have confirmed that an EAT26 score ≥20 should prompt the need to explore substance use [21], [ 22], and DSM and EAT26 criteria are concordant in identifying a greater risk of substance abuse, affecting bulimic (rather than restrictive) profiles. A very recent study in 1800 students shows that an EAT26 score ≥20 doubles the occurrence of cocaine and tranquilizer consumption, and augments that of alcohol by ∼30%, especially in females [23]. Our data extend these findings to a much larger sample of Italian adolescent students. The correspondence between EAT26 ≥20, overweight and drug use was higher in Central regions, than in Northern and Southern regions. This may be due to a different degree of family control and economic dependence, which are stronger in the South than the North of the country, likely favouring a more isolated development of obesity in the former and of drug use in the latter.

Along with the greater consumption of cocaine, hallucinogens, and heroin, we observed a lower consumption of cannabis in the two opposite extreme percentiles of the BMI distribution. This may be related to the perceived weight-promoting effects of this kind of substance, or to social self-emargination since marijuana is typically consumed in gathering situations. Compared with normal- weight individuals, a lower percentage of under- and overweight adolescents reported having drug abusing friends, suggesting that peer-to-peer influences do not justify the greater use of drugs observed in these subjects. Overall, our data indicate that both obesity and underweight are associated with a habitual use of drugs in Italian adolescents, thus extending to this population the evidence that novelty seeking and impulsivity may be common underlying factors [20]. In this line of reasoning, it has been suggested that the attempt to refrain from eating, which is typical in both underweight and overweight subjects, increases the risk of compensatory substance use, which in turn may influence food intake [20]. Accordingly, animal studies have shown increased self-administration of ethanol, alcohol and cocaine under conditions of food deprivation [24]–[26], and human studies have shown positive associations between the severity of dieting and the prevalence of alcohol, cigarette, and drug use both in overeaters and in at-risk-dieters [27].

### Regarding the compelling need to identify preventable risk elements, how strong is the social, potentially modifiable component underlying the above disorders?

The multinomial analyses clearly pointed out that, once accounting for psychosocial factors, the relationship between overweight and illegal drug consumption was fully abolished. This may be taken as to indicate that the psychosocial environment underlying overweight and drug consumption is similar, and therefore responsible for their correlation. Our data indicate that satisfaction with self and friendships were inversely, and sedentary habits were directly associated with overweight, along with avoiding going out with friends. Consistently, in a smaller sample (n = 268) of 16-year-old students attending high school, lower self-esteem was found to be related with higher scores on the EAT26 and with health-risk behaviours, including cigarette smoking, alcohol use, and drug use [28]. The present sample also showed that lower parental schooling and closer maternal relationships potentially contributed to the association of food and drug seeking. Though associations do not prove causality, it is a fact that parental schooling must precede the occurrence of obesity in the offspring, and that a lower educational level is usually more common in the Central-Southern Regions of Italy, where overweight was more prevalent. Fathers' occupation, reflecting the level of education, was similarly related to childhood obesity in French girls [29]. Binge drinking in both genders and cigarette smoking in males remained associated with overweight, and it is of interest that binge drinking may trigger a food binge [30], thus promoting overweight. Alcohol consumption and cigarette smoking may also serve as complementary non-illegal sources of reward, along with the habit of spending a great deal of money without parental control, playing videogames and watching more than 4 hours of TV/day, as observed here.

Notably, the use of diet products and EAT26 score remained related to overweight regardless of adjustment for any personal, family or leisure time factor, suggesting that none of these factors reinforced or weakened the likelihood that overweight adolescents would develop an eating disorder. Similarly, none of the above mentioned variables modified the relationship between underweight and drug use, remaining positive for cocaine in females and hallucinogens in males. The selection of a specific drug (in this case cocaine) has been suggested to serve to achieve and maintain the desired social standard in body weight, especially in female adolescents who are more easily influenced by the current cultural environment [31]. A recent study in Swedish females showed that having highly educated parents and higher school grades had an augmented risk of being hospitalized for an eating disorder, possibly due to demanding internal and external expectations [32]. We observed a tendency towards higher levels of parental schooling and self-esteem in underweight subjects, supporting the possibility that parents transmit to their offspring the perception that being slim increases social acceptance.

### What are the clinical implications of the current findings?

The importance of the current findings lies in the potential overlap in the damage to body organ systems provoked by co-existing risk conditions. Overweight has been linked to multiple health problems in children and adolescents, including glucose intolerance and cardiovascular risk factors, predicting a spread in disease occurrence during adulthood [33]–[35]. Eating disorders have long-term consequences for several organ systems [36], and they are associated with the highest mortality rate among psychiatric disorders; much of the mortality and morbidity stems from cardiovascular complications [37].

Our data indicate that at least one-quarter of normal-weight adolescents carry the risks associated with recent drug use, and 25% in the overweight category carries both the risks related with drug consumption and those associated with overweight. Our co-morbid (overweight plus drug taking) adolescents were characterized by specific psychosocial traits, including a history of regrettable sexual intercourse, serious problems with parents and truancy from school. This is consistent with a previous meta-analysis, identifying childhood sexual abuse, lack of parental support and difficulties at school as risk factors for obesity [38]. It was shown that repeated activation of stress centres may lead to ‘stress eating’ and over-eating [39], [ 40].

One very important clinical outcome of this study is the demonstration that the association between overweight and drug use is explained by personal and family variables. These are acquired and modifiable, not innate risk factors, entailing the possibility for intervention and prevention.

In conclusion, the use and the abuse of prohibited substances are 20–40% greater in abnormal- than normal-weight adolescents. The frequent association of overweight and substance use and the demonstration of a common underlying social, family and personal maladaptation (ranging from dissatisfaction with school performance, friendship, and parental care, to serious problems with parents and regrettable sexual intercourse) underlines the need to better delineate an interdisciplinary approach involving individual-focused treatment models as well as public health, social and environmental change to reduce food and substances-related problem.
